# The thermoplastic techniques or single-cone technique on the quality of root canal filling with tricalcium silicate-based sealer: An integrative review

**DOI:** 10.4317/jced.59387

**Published:** 2022-07-01

**Authors:** Caroline-Felipe-Magalhães Girelli, Mariane-Floriano-Lopes-Santos Lacerda, Cleidiel-Aparecido-Araújo Lemos, Mariana-Reis Amaral, Carolina-Oliveira Lima, Frank-Ferreira Silveira, Eduardo Nunes

**Affiliations:** 1 MSc, Department of Dentistry, Pontificial Catholic University of Minas Gerais, Brazil; 2 DDS. Department of Dentistry, Juiz de Fora Federal University, UFJF/GV- MG; 3 DDS. Department of Dentistry, Pontificial Catholic University of Minas Gerais, Brazil

## Abstract

**Background:**

The filling of the root canal system (RCS) is an important step in endodontic treatment and aims to obtain a three-dimensional sealing of the root canal spaces to prevent bacterial contamination. For this, the selection of an appropriate sealer must be performed synchronously with the choice of the root canal filling technique. This study aims, through an integrative review, to evaluate the quality of root canal filling by comparing thermoplastic and single-cone (SC) techniques.

**Material and Methods:**

The Medline/PubMed, Scopus, Web of Science and Virtual Health Library (VHL) databases were used to find articles published until November 2021. The eligibility criteria comprised articles that evaluating the quality of root canal filling comparing thermoplastic techniques with the SC technique using tricalcium silicate-based sealer. Studies that evaluated primary teeth, endodontic retreatment or perforations, different outcomes, and studies that considered artificial teeth or different sealer and material for obturation of different techniques were excluded. For articles that were not available for access, an additional contact with authors were considered. A total of 1699 articles were found. After duplicate removal, the title and abstract of 828 articles were screened. Sixteen articles were considered for full-text analysis, but only ten articles met the eligibility criteria. Data extracted from the studies were discussed and tabulated to allow the comparison of desired factors.

**Results:**

Concerning the formation of gaps/voids, the thermoplastic techniques showed better results than the SC technique in 3 articles. On the other hand, 2 articles reported no statistical difference between the tested techniques. In addition, about the penetration of tricalcium silicate-based sealer in the dentinal tubules, of the 5 articles selected, in 4 there was no significant difference between the tested techniques and only one study showed better penetration of the sealer when using thermoplastic techniques.

**Conclusions:**

The thermoplastic technique was better in most selected studies regarding gaps and voids, but regarding the penetration of the sealer into the tubules, both techniques were effective.

** Key words:**Root canal filling, thermoplastic techniques, tricalcium silicate.

## Introduction

The aim of root canal filling is to achieve the three-dimensional sealing of the root canal spaces to obtain a fluid-tight barrier, stable over time, for preventing bacterial contamination or recontamination, enabling conditions for the repair of periradicular tissues (1-3). For this, the selection of an appropriate sealer and the root canal filling technique is mandatory (4-6).

Cold lateral compaction is the most commonly filling technique worldwide and is regarded as the standard against which other filling techniques. Despite being predicTable and relatively simple to execute in regularly tapered canals, root canal filling using a lateral using a lateral compaction technique might lack homogeneity and thus result in a great amount of sealer (4).

Thermoplastic techniques (continuous wave of condensation (CWC), Tagger’s hybrid technique (THT), carrier-based (Thermafill system) have been developed to incorporate the use of thermal or frictional heat to obtain gutta-percha thermoplastic molding (2-6) to allow advantageous results for the management of irregular shaped root canals, allowing better adaptation to the canal walls, with a more homogeneous filling (6,7).

Similarly, the single-cone technique (SC) has become used because it is easier to implement, consequently it is less sensitive to operator variations, has low cost, and short operation time. This technique uses a gutta-percha cone with a diameter similar to the last instrument used to shape the root canal. However, this technique demands a greater amount of sealer and thus, the flowability and other physicochemical properties of the sealer play an essential role in the success of the endodontic treatment (3,7).

Thereby, tricalcium silicate-based sealers have attracted considerable attention because of their high biocompatibility, slight expansion while setting. Moreover, this sealerhas high bioactivity and canpromotes chemical adhesion between the dentin walls and the filling material, through the formation of a structure similar to the biological hydroxyapatite (8-11).

To date, there is no consensus in the literature on which root canal filling technique should be chosen when using the tricalcium silicate-based sealer, to improve the quality of the root canal filling. Furthermore, due to the growing amount and complexity of information in the health area, it has become essential to develop devices, in the context of scientifically based research, capable of delimiting more concise methodological steps and providing professionals with better use of the elucidated evidence in numerous studies. In this scenario, the integrative review emerges as a methodology that provides the synthesis of knowledge and the incorporation of the applicability of results of significant studies in practice.

In order to synthesize the results obtained in research on this topic comprehensively, the integrative literature review provides broader information on the subject/problem (12). Therefore, the aim of this study was, to compare through an integrative review, the thermoplastic techniques and the SC technique as the filling quality (formation of gaps and sealer penetration in dentinal tubules) when used with a tricalcium silicate-based sealer.

## Material and Methods

This study selected the integrative review method to achieve the proposed objective of comparing two or more root canal filling techniques, one of which was the thermoplastic technique and another which was a SC technique when associated with tricalcium silicate-based sealer in the filling quality of the RCS. The question that supported the scientific evidence collection was: “Which root canal filling technique (SC or thermoplastic techniques) promotes better filling quality when combined with tricalcium silicate-based sealer?”. An electronic search was performed in the online databases MEDLINE/PubMed (database developed by the National Center for Biotechnology Information, National Library of Medicine), Scopus, Web of Science, and Virtual Health Library- VHL (Lilacs and BBO). The same units were considered, however, due to the particularities of the databases, as the searches were performed according to Table 1.

**Table 1:**
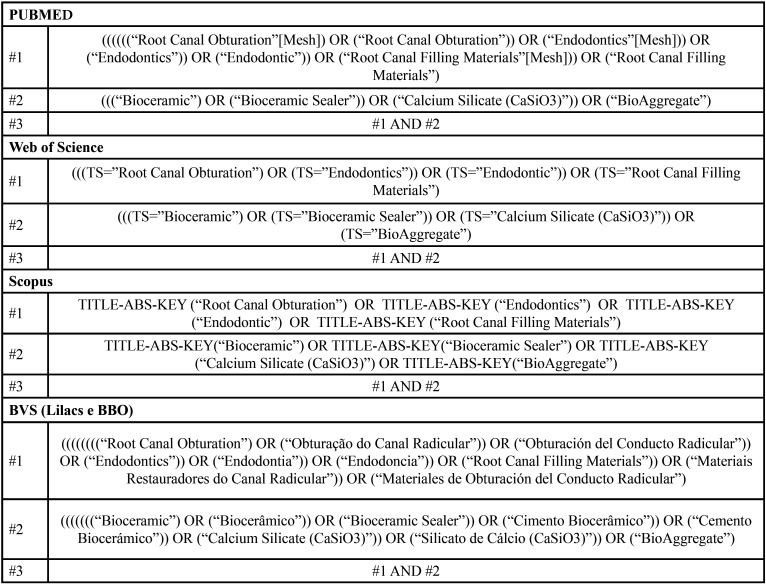
Search strategy used for each database.

To carry out the selection, the Rayyan QCRI reference manager was used in order to determine the eligible potential for this integrative review. At first, studies dealing with duplication of residues between databases were identified and excluded.

Thus, the selection was performed after reading the title, abstract, and keywords. This was done independently by two reviewers (C.F.M.G and M.R.A). After resolution of any ambiguity by a discussion with a third reviewer (M.F.L.S.L), the studies identified during the initial search were independently evaluated by both reviewers with full-text reading. In addition, a manual search was performed in the reference lists of eligible studies, as well as a cross-search in the authors’ database, checking the possibility of selecting new relevant articles. For articles that were not available for access, an additional contact with authors of the published studies were considered.

The eligibility criteria were: articles published until November 2021, and that their content addressed the following criteria for evaluating the quality of root canal filling (sealer penetration in dentinal tubules, or/and voids/gaps formation), comparing thermoplastic techniques with the SC technique when associated with tricalcium silicate-based sealer.

On the other hand, the exclusion criteria comprised studies performed on primary teeth, which addressed the use of tricalcium silicate-based sealer in endodontic retreatment or perforations. Studies that evaluated apical leakage using the dye extraction leakage method, and when the bond strength of the root canal filling materials was evaluated by the push-out test or sealer penetration in simulated lateral canals were also excluded. In addition, studies that considered artificial teeth or different sealer and material for obturation of different techniques were excluded.

Data extracted from the studies included, author/year, the tricalcium silicate-based sealer used, thermoplastic technique used, compared technique, measurement method, evaluation method, and the results. The data were extracted by two reviewers independently, discussed, and tabulated. The details were tabulated to allow comparison between the studies regarding the completeness of obturation (Table 2).

**Table 2:**
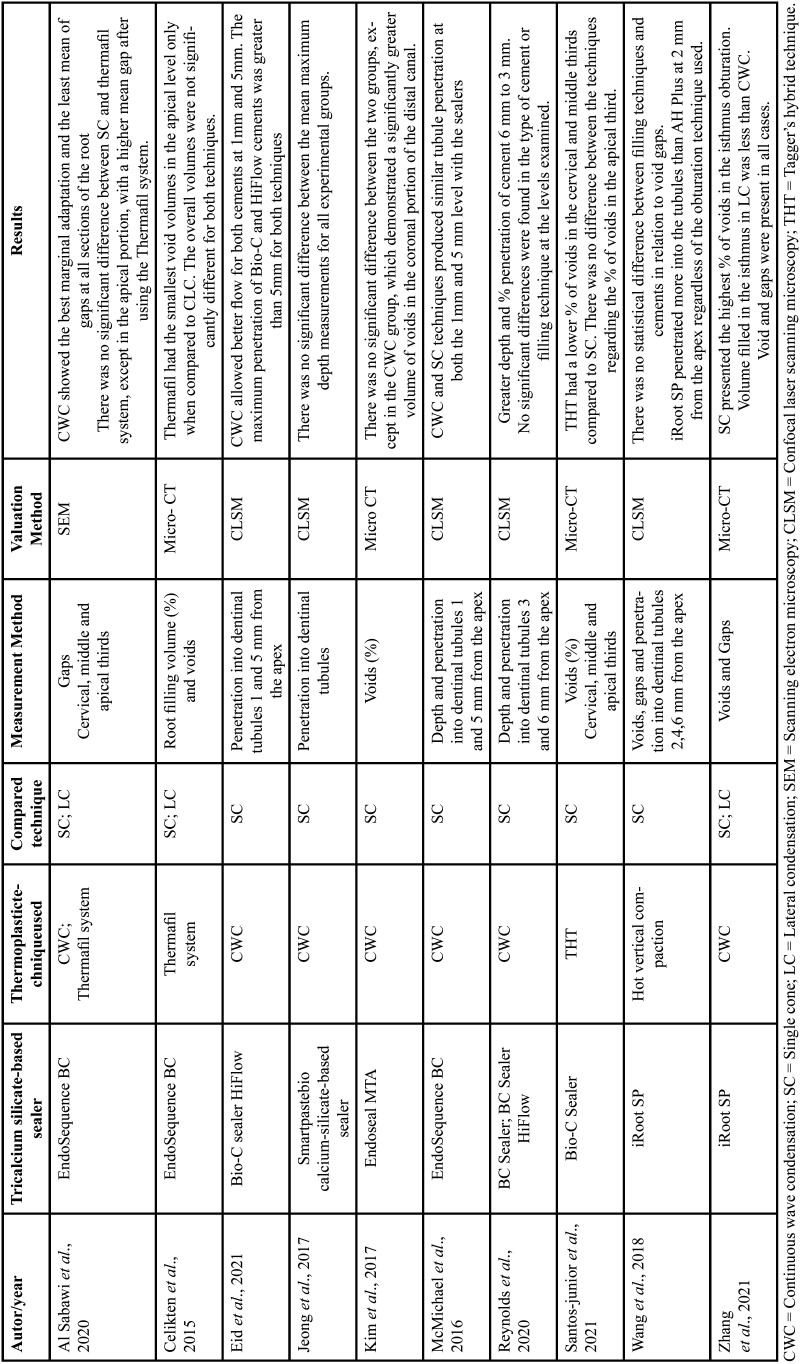
Data extracted from selected studies.

Reviewers assessed the studies based on the same criteria. For each selected study, sample numbers, tabulated root canal filling methods, and efficacy of the established protocol. The combination of these factors provided a new set of articles that were used in the present review.

## Results

**Figure 1:**
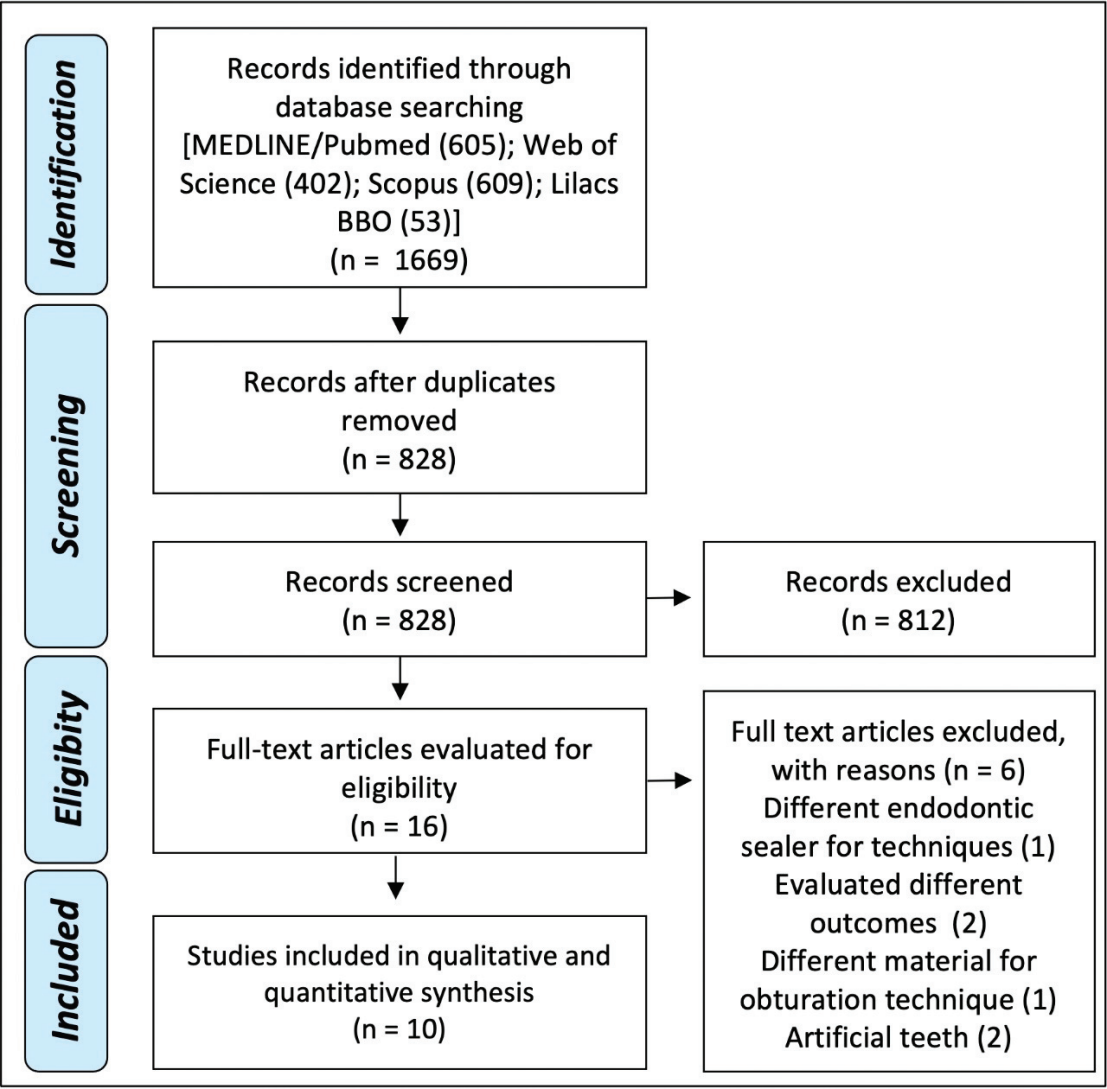
Flow diagram of bibliographic research.

An initial search of databases using the search terms revealed a total of 1699 articles in the databases. After the removal of duplicates, 828 articles were screened using the titles and abstracts. After reading the full texts, a total of 16 articles were selected for. Out of these articles, 6 articles did not satisfy the inclusion criteria, because three articles evaluated different endodontic sealers for both evaluated techniques (2,13,14), two studies evaluated different outcomes (bond strength and apical leakage) (15,16) and one study considered the evaluation in artificial teeth (17). Therefore, 10 articles were included in the review (Fig. 1).

Of the selected articles, all were published in dental journals, between 2015 and 2021. Analyzing the void/gap formation, of the 5 articles selected, the thermoplastic technique showed better results than SC technique in three articles and no statistical difference between the techniques tested in two selected articles. Regarding the penetration of tricalcium silicate-based sealer in the dentinal tubules, of the 5 selected articles, 4 there was no significant difference between the tested techniques, and only one article showed better penetration of the sealer in the dentinal tubules with the use of the thermoplastic technique.

## Discussion

A successful filling of the RCS is directly related to the absence of empty spaces between the filling material and the dentin walls (gaps), as well as inside the filling mass (voids) (5,18,19). Non-obturated areas may allow bacteria to remain at a site and lead to treatment failure. Furthermore, proper root canal filling is extremely important to prevent the migration of bacteria and their products to the periradicular tissues, potentially leading to reinfection (1,5,19). 

The present study compared the thermoplastic techniques and SC technique associated with tricalcium silicate-based sealer, concerning the root canal, filling quality, through an integrative review. For this purpose, the volume of voids/gaps was used as a method of assessing root canal filling quality.

The continuous wave of condensation (CWC) has the least means of gaps at all sections of the root when compared with SC technique (20). In addition, the flattened root canals filled using the tagger’s hybrid technique (THT) created a smaller percentage of voids in the cervical/middle thirds than those filled using the SC technique (7). These facts can be attributed to the characteristics of thermoplastics techniques which promote a more homogeneous filling mass with good adaptation to the canal walls, as well as greater penetration into the lateral canals, isthmus, and recess (4-7,20).

The continuous wave of condensation (CWC) has fewer gaps at all sections of the root when compared with SC technique (6,20). In addition, the flattened root canals filled using the tagger’s hybrid technique (THT) created a smaller percentage of voids in the cervical/middle thirds than those filled using the SC technique (7). These facts can be attributed to the characteristics of thermoplastics techniques which promote a more homogeneous filling mass with good adaptation to the canal walls, as well as greater penetration into the lateral canals, isthmus, and recess (4-7,20). On the other hand, the SC technique lacks vertical and lateral pressure during the obturation procedure and without pressure, it is difficult for the filling materials to enter the isthmus regions, therefore, there was a possibility of more and larger voids forming in the isthmus in the SC technique (1,6).

On the other hand, it was also possible to identify the absence of a significant difference in the percentage of empty spaces between CWC and SC techniques in other studies (1,21). This result can be attributed to the mechanism of action of the tricalcium silicate-based sealer, which is described as a chemical reaction between the dentin wall and the silicates of the filling material, through a hydration reaction, leading to the formation of hydroxyapatite, which can attenuate the spaces at the dentin/filling material interface and minimize the overall incidence and size of spaces (8-10,12,21-23). In addition, tricalcium silicate-based sealers have the ability to mitigate important disadvantages of the CS technique when the gutta-percha cone does not adapt to irregularities of oval canals and isthmuses. A filling sealer without chemical properties and adhesiveness to dentin can accumulate in these areas resulting in poor marginal adaptation, porosity, fixation contraction and voids (15).

To achieve tubule penetration, the particle size of the material must be smaller than the tubule diameter; the larger the tubule, the deeper a particle can penetrate. The penetration of the endodontic sealer forms a physical barrier with the dentin, improves filling retention and confine residual bacteria (4). However, the sealer penetration depth into the dentinal tubules is affected by several factors, such as the physical and chemical properties of the sealers, the effectiveness of smear layer removal, the anatomy of the RCS and the filling technique (4). Based on the results of selected studies, the capacity of the filling material to penetrate the dentinal tubules did not show significant difference when using CWC or SC techniques for the penetration of tricalcium silicate-based sealer into the dentinal tubules (24-27). This result may be associated with fine particles of tricalcium silicate-based sealers (<1 μm) (4,7,19,21,28).

Thus, tricalcium silicate–based sealers penetrated into the dentinal tubules without applying intracanal compaction pressure that is usually used in thermoplastics techniques. This finding would be clinically significant because it would eliminate the application of excessive forces that could cause root fractures, especially in thin roots. Regarding the relationship between the penetration of the tricalcium silicate-based sealer in the dentinal tubule and sealability, it is only possible to infer that there is a chemical bond at the sealer and dentin wall, but further studies need to be carried out to know whether it could prevent or minimize bacterial recontamination.

Regarding the methods for evaluating the quality of fillings, micro-CT proved to be a favorable option to calculate empty spaces in both techniques for root canal fillings. This non-destructive and highly accurate method provides 2D scans and 3D reconstructions at different grayscale levels allowing to distinguish all the different components of root canal filling, such as gutta-percha, sealer, gaps, and voids (3,5,18). However, other methods such as scanning electron microscopy and confocal laser microscopy were also effective in other measurements such as sealer penetration into the root canal in dentinal tubules and analysis of the morphology of the sealer’s ultrastructure on the root canal wall in different levels of cut (20).

The integrative review has some limitations, such as the fact that it includes studies with different methodologies, which can difficult the synthesis of the results and and possible bias (3). Thus, to reduce this facts, the authors made an effort to establish a rigorous eligibility criteria like teeth filled with bioceramic, use of techniques thermoplasticized and SC.

Based on the results of this integrative review, it is not possible to accurately conclude which technique is better, given the variation between the tricalcium silicate-based sealers used and the various thermoplasticizing techniques, which may interfere with the quality of root canal filling. However, based on the above, it was possible to observe that thermoplastic technique showed best results in most selected studies regarding gaps and voids, but regarding the penetration of the sealer into the tubules, both techniques were effective.
